# The Effect of Plant Growth Compensation by Adding Silicon-Containing Fertilizer under Light Stress Conditions

**DOI:** 10.3390/plants10071287

**Published:** 2021-06-24

**Authors:** Natalya A. Semenova, Alexandr A. Smirnov, Andrey A. Grishin, Roman Y. Pishchalnikov, Denis D. Chesalin, Sergey V. Gudkov, Narek O. Chilingaryan, Anastasia N. Skorokhodova, Alexey S. Dorokhov, Andrey Y. Izmailov

**Affiliations:** 1 Federal State Budgetary Scientific Institution “Federal Scientific Agroengineering Center VIM” (FSAC VIM), 109428 Moscow, Russia; natalia.86@inbox.ru (N.A.S.); as984788@gmail.com (A.A.S.); 5145412@mail.ru (A.A.G.); narek-s@list.ru (N.O.C.); dorokhov.vim@yandex.ru (A.S.D.); vim@vim.ru (A.Y.I.); 2 Prokhorov General Physics Institute of the Russian Academy of Sciences, 119991 Moscow, Russia; genoa-and-pittsburgh@mail.ru (D.D.C.); s_makariy@rambler.ru (S.V.G.); 3 Institute of Biology and Biomedicine, Lobachevsky State University of Nizhni Novgorod, 603022 Nizhni Novgorod, Russia; 4 Moscow Timiryazev Agricultural Academy, Russian State Agrarian University, 127550 Moscow, Russia; red-green216@mail.ru

**Keywords:** light-emitting diode, sodium lamps, plants cultivation, silicon fertilizer, red-leaved lettuce, green-leaved lettuce

## Abstract

The effects of different spectral compositions of light-emitting diode (LED) sources and fertilizer containing biologically active silicon (Si) in the nutrient solution on morphological and physiological plant response were studied. Qualitative indicators and the productivity of plants of a red-leaved and a green-leaved lettuce were estimated. Lettuce was grown applying low-volume hydroponics in closed artificial agroecosystems. The positive effect of Si fertilizer used as a microadditive in the nutrient solution on the freshly harvested biomass was established on the thirtieth day of vegetation under LEDs. Increase in productivity of the red-leaved lettuce for freshly harvested biomass was 26.6%, while for the green-leaved lettuce no loss of dry matter was observed. However, being grown under sodium lamps, a negative impact of Si fertilizer on productivity of both types of plants was observed: the amount of harvested biomass decreased by 22.6% and 30.3% for the green- and red-leaved lettuces, respectively. The effect of using Si fertilizer dramatically changed during the total growing period: up to the fifteenth day of cultivation, a sharp inhibition of the growth of both types of lettuce was observed; then, by the thirtieth day of LED lighting, Si fertilizer showed a stress-protective effect and had a positive influence on the plants. However, by the period of ripening there was no effect of using the fertilizer. Therefore, we can conclude that the use of Si fertilizers is preferable only when LED irradiation is applied throughout the active plant growth period.

## 1. Introduction

Considering environmental factors in agriculture, light is one of the most important ingredients influencing plant growth, development, and production. It is known that most of Russia’s territory, particularly its northern regions, suffers from a lack of sunlight, which is needed to maintain a high level of plant production during winter periods. For this reason, the deficiency of natural sunlight is usually compensated by using supplementary assimilation lamps in greenhouses. For instance, high-pressure sodium lamps are a very common additional lighting source. Despite well-established greenhouse technologies, the development of LED lighting systems has proved to be a subject of considerable attention over the last decade [1,2]. Above all, the efficiency of LEDs is higher compared to the sodium lamps; moreover, the potential design and optimization of LED lighting systems are rather flexible. Due to low energy emission, a light source can be located in close proximity to or within the lampshade [3]. Since LEDs emit in a narrow spectral range, any combination of diodes of different colors can be applied to control plant growth and development. Nevertheless, to consider LEDs as a valuable light source for greenhouse technology and horticulture, it would be helpful to conduct quantitative studies on plant responses to LEDs of different spectral ranges [4].

The absorption spectrum of a green leaf is characterized by three pronounced frequency ranges: the 300–400 nm high-energy range corresponds to the Soret band of chlorophylls; 400–550 nm is the region of carotenoid absorption; and 600–800 nm is a region of intense absorption of the Qy electronic transition of chlorophylls [5]. Light quanta of other spectral regions are also absorbed by plants through photoreceptors that stimulate specific developmental processes [6]. Combinations of incident lights from the 300–800 nm range affect plant morphology and can cause some changes in flowering and flower color [7]. Absorption in the red region drives basic photosynthesis processes, which is why in horticulture the most commercially used light sources are red ones. Generally, red light stimulates the growth of branches and bud outcome. Green light corresponds to the low-energy part of the green leaf spectrum, and its possible influence on photomorphogenesis is still under debate. It is assumed that green light can penetrate deeper into the leaf, increasing the light absorption in lower leaf layers, and, therefore, the intensity of photosynthetic processes. It has been reported that, with an increase in the proportion of green light, the dry mass of lettuce is also increased [8]. On the other hand, there are some studies that report no pronounced effects of green light or unconvincing results [9,10]. Blue light is essential for normal functioning of plants. Only about 10% of blue light is needed to prevent any photosynthetic dysfunction caused by its lack in lighting. Blue light sources can be used additionally to improve growth and prevent unwanted effects such as excessive stem elongation. Thus, it is obvious that variations of intensities of the irradiation spectrum can control the photomorphogenic response of plants and might significantly enhance crop production [11,12,13].

Besides variations in light conditions, different types of fertilizers can be used to improve plant growth and production, particularly silicon-containing fertilizers [14,15,16,17,18,19,20]. Si is one of the most abundant elements on Earth; its concentration corresponds to 14–20 mg Si/L [21,22], which does not go beyond the concentrations of other inorganic elements. Since plants take up Si and transfer it from roots to shoots in the form of H_4_SiO_4_ [23], any soil can be classified by the availability of soluble Si. Many studies have revealed that Si actively moderates morphological and physiological responses in plants [21,24,25,26]. It has been shown that by using Si fertilizers the number of foliar and soilborne diseases can be significantly decreased in many agricultural crops [27,28,29,30]. Moreover, the efficiency of some photosynthetic processes involved in the regulation of antioxidant mechanisms is improved for plants grown with Si fertilizers [21].

In this study we assess the combined effect of different spectral ranges of irradiation and using Si fertilizers during the growing period of red- and green-leaved lettuces [31,32,33,34,35,36,37]. Considering various light types used in our study as sources of stress conditions, Si nutrient solution was used to explore a possible effect of stress compensation. Working with two climatic chambers allowed us to grow plants simultaneously under sodium lamps and under LEDs. Two regimes of lighting in the LED climatic chamber were employed: one was for cultivation of lettuce before the massive appearance of shoots (first five days), and the other was for the subsequent cultivation of lettuce up to the ripeness of the product. Varying the amount of Si fertilizer throughout cultivation, we estimated the effect it has on the cultivation of the plants.

## 2. Materials and Methods

### 2.1. Climate and Light Conditions

**Figure 1:**
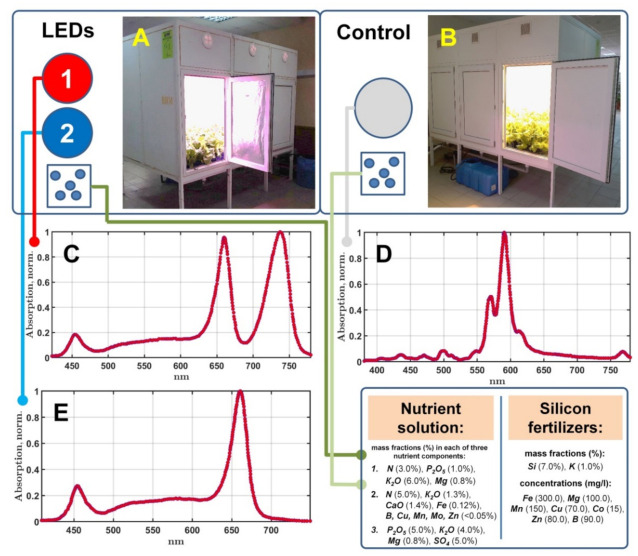
Scheme of the experimental setup. Two climatic chambers equipped with LEDs (**A**) or sodium lamps (**B**) and the corresponding spectra of the lighting regimes are shown in plots (**C**–**E**).

All experiments were carried out in two climatic chambers developed in the Federal Scientific Agroengineering Center VIM, Russia (Figure 1A,B). The chambers are of the same size–2500 × 1600 × 1700 mm. Each chamber has only one level for plant cultivation. The maximum plant height is 1500 mm. The total usable area for growing was 3.8 m^2^. The total power consumption was 3 kW. The intensity of light was measured by a TKA-VD spectrocolorimeter (TKA Scientific Instruments, Saint-Petersburg, Russia) at 15-cm height in each climatic chamber.

Climatic chamber 1 (Figure 1A) is equipped with a system of lighting based on LEDs, and it has two spectral regimes of irradiation. The first regime of lighting is characterized by the visible spectrum shown in Figure 1C. It was applied at the earlier stage of plant growth (the first 5 days) and has the distribution of photosynthetic phonon flux density (PFD) over the frequency ranges as shown in Table 1. The second regime of lighting was applied after 5 days of cultivation and was intended to stimulate the reverse response of phytochromes. This is achieved by reducing the intensity in the red region of the LED spectrum (Figure 1E). The PFD distribution for this regime is shown in Table 1.

Lighting in the second climatic chamber (Figure 1B) was provided by two tubular sodium lamps (yellow color) and one metal halide lamp (white color). The averaged PFD of the chamber is 132.3 μmol m^−2^ s^−1^. The temperature settings in both climatic chambers at the first stage of cultivation were 19–20 °C during the day and 16–18 °C during the night; those of at the second stage of cultivation were 20–22 °C and 18–20 °C, respectively. The relative humidity in the chambers was maintained at 65–70% throughout the experiments. The temperature of the nutrient solution was 18–20 °C.

Preparation of the climatic chambers for sowing the seeds was as follows: (1) sanitization of the inner and outer surfaces of the chambers; (2) equipping the chambers with containers of mineral wool used as a substrate for the plants; (3) setting up the spectral composition of LEDs corresponding to the initial stage of cultivation; (4) regulation of the automatic watering system for the plants.

### 2.2. Cultivation of the Plants

Ten containers filled with the chemically inert substrate (mineral wool), which is highly permeable to water and has high water-holding capacity, were installed in each climatic chamber. The mineral wool in the container was divided into two rows, each of which contained five square nests. In each nest, five small depressions were made in which one seed of the red- or green-leaved lettuce (*Lactuca sativa* L.) was sown [38]. Such types of salad lettuces are commonly used to perform diverse studies on plant cultivation under different conditions [39,40,41,42], We experimented with two varieties of lettuce: the green-leaved lettuce called Azart (Prestizh Semena, www.pr-semena.ru, accessed on 1 May 2020, Russia), and the red-leaved lettuce called Robin (MoravoSeed, 1 May 2020, Czech Republic).

Before sowing the seeds, the mineral wool was prepared by completely moistening it with the nutrient solution, the concentration of which was 10% lower than that of the main solution. The nutrient solution is based on the FloraSeries fertilizer kit (GHE), which provides all the necessary macro and micronutrients. The kit contains FloraGro, FloraMicro SW, and FloraBloom components. We used the following ratio of the components: 2.5:1.6:1. The corresponding mixture of hydroponic kits was chosen according to the recommendations of the fertilizer manufacturer (generalhydroponics.com, 4 December 2019), taking into account the specificity of our research (focusing on green mass). Particularly, at the phase of seed germination the concentration of hydroponic kits in the solution was less than at the phase of vegetative growth. FloraGro supplement promotes structural growth and gain of active green mass. This supplement provides plants with a sufficient amount of nitrogen and potassium, as well as secondary minerals: 1.0% of ammonia nitrogen, 2.0% of nitrate nitrogen, 1.0% of phosphorus P_2_O_5_, 1.0% of soluble potassium K_2_O, and 0.8% of magnesium. FloraMicro helps to stabilize the pH level of the nutrient solution. It contains 1.5% of ammonia nitrogen, 3.5% of nitrate nitrogen, 1.3% of soluble potassium, and the following EDTA-chelated microelements: Cu (0.01%), Mn (0.05%), Zn (0.015%); EDDHA and OPTA chelated Fe (0.12%) and Mo (0.004%). FloraBloom supplies plants with an adequate amount of phosphorus and potassium, which are involved in bud and fruit formation. The content of microelements in this supply is the following: P_2_O_5_ (5.0%), K_2_O (4.0%), Mg (3.0%), SO_4_ (5.0%).

### 2.3. Silicon Fertilizer

A liquid silicon-containing fertilizer was used in the experiments. The preparation is composed of silicon and potassium with mass fractions Si (7.0%) and K (1.0%) and trace elements in easily available chelate forms for plants (mg/L): Fe (300.0), Mg (100.0), Cu (70.0), Zn (80.0), Mn (150.0), Co (15.0), B (90.0).

### 2.4. Biometric and Biochemical Analysis

To determine the impact of different light conditions and fertilizer treatment, careful selection and preparation of plant samples were made. Leaves of the selected samples (for plants that have not reached the stage of forming flower stalks) were fresh, healthy, and undamaged. Their shape, color, and smell corresponded to their botanical type and variety. The leaf surface was not damaged by pests or their waste products. The assimilating leaf surface showed no sign of excessive external moisture.

The lettuce was sampled by taking one plant from each container and then cutting a rosette at the base of the plant. All samples were placed in hermetically sealed bags for further study. The total number of samples was 25 plants for each type of experiment (variety, lighting, fertilizer). Twelve plants were used to determine morphological parameters and 13 plants to determine their photosynthetic parameters. Morphological parameters (weight, number, and area of leaves) were determined for each selected plant. The biometric parameters of the morphological organs of the plants by the phases of their development were assessed 15, 30, and 45 days after the lettuce shoots appeared.

To determine the mass of dry matter in the plants, a sample was crushed manually using a hand cutting tool until the fragment size of no more than 1 cm. After grinding, the sample was mixed to avoid the inhomogeneity of fragments. Then, two weighed portions with mass of at least 5 g were isolated in two replicates using an analytical balance. The samples were dried in an oven for either 3 h at 60–70 °C or 1 h at 105 °C.

To estimate the moisture in fresh green leafy mass, the test sample was crushed and mixed, and two portions of 25–50 g were weighed on a balance with an accuracy of 0.001 g. Then the portions were placed in weighing bottles pre-dried to a constant weight. The containers with weighed portions were placed for 20–30 minutes in an oven heated to 120–130 °C to inactivate enzymes and then dried at 105 °C to constant weight.

The quantitative analysis of pigments (chlorophylls, carotenoids) included their extraction from the plant tissues using acetone, separation of the mixture into individual components, and spectrophotometry.

The concentrations of pigments in 100% acetone were calculated according to Holm–Wettstein as follows:

$$C_{Chl_{a}}=9.784\times D_{662}-0.990\times D_{644}$$

$$C_{Chl_{b}}=21.426\times D_{644}-4.650\times D_{662}$$

$$C_{Chl_{a}+Chl_{b}}=5.134\times D_{662}+20.436\times D_{644}$$

$$C_{car}=4.695\times D_{440.5}-0.268\times C_{Chl_{a}+Chl_{b}}$$

Here $C_{Chl_{a}}$, $C_{Chl_{b}}$, and $C_{car}$ are the concentrations of chlorophylls *a* and *b* and the carotenoids. $D_{\omega }$ is the optical density of the extract at the corresponding wavelength ω in nm.

To determine nitrate ion concentration, a 1% solution of aluminum-potassium alum was used as the extraction solution. The concentration of nitrate ions was measured using an NO_3_-selective electrode connected to an Ekspert-001 ionometric station (Econiks, Russia). The station was calibrated using solutions containing a known concentration of KNO_3_ (Sigma-Aldrich, SL, USA).

Vitamin C in the leaves of lettuce plants was determined by a spectrophotometric method with a dye solution of 2,6-dichlorophenol indophenol [43]. The sucrose concentration was determined by refractometry [44].

### 2.5. Statistical Analysis

The biometric and biochemical parameters were processed by applying ANOVA. To estimate the statistical significance of the considered parameters, the F-test and the least significant difference test were applied.

## 3. Results

### 3.1. Effect of Fertilizers and Lighting on the Productivity of Lettuces 15 Days after the Emergence of Mass Shoots

At the initial stage of growth (up to 15 days), lettuce exposed to light stress (chamber 1) lagged behind in growth, and the addition of Si fertilizer to the nutrient solution had a depressing effect in all variants of the experiment for both lettuce varieties (Table 1).

Analysis of variance of the data showed a significant effect of factors such as lighting and the use of Si fertilizer on the fresh weight of both lettuce varieties. Under the sodium lamps, lettuce of both varieties developed faster, and by the 15th day of cultivation the increase in fresh weight was about 53%, LSD = 0.26 (Table S1). This effect is caused by light stress during the germination phase.

The use of Si fertilizer oppressed the plants regardless of the variety and the lighting. The average loss of fresh weight was 18.5% (Table 1). All three factors (lighting, variety of lettuce, Si fertilizer) had a significant impact on the number of lettuce leaves (Table S1). Under sodium lamps, the number of leaves of both varieties is on average 1 pc. more (by 21%) than under LED irradiation, LSD = 0.21 (Table S1). Also, Si fertilizer negatively influenced the number of leaves in the first stage of development of both varieties of lettuce regardless of the type of lighting. Independent of other factors, the red lettuce produced more leaves than the green lettuce.

A significant effect of lighting and the use of Si fertilizer on the dry weight of lettuce of both varieties was revealed. The dry weight of the lettuce changed significantly depending on lighting, and the reaction of the plants was variety specific. For red lettuce (Robin), the best results on the accumulation of dry matter were observed under LED irradiation, while for the green lettuce (Azart) it was under that of sodium lighting.

The area of the leaf surface when illuminated with sodium lamps was 69% greater than under LED lighting. Generally, the use of Si fertilizer caused a decrease in the photosynthetic surface of plants by about 43.5%. However, the largest significant difference of the leaf surface area was observed under sodium illumination without the addition of Si fertilizer.

Analysis of pigment concentrations showed that the red lettuce, regardless of the influence of other factors, accumulated 10% more chlorophyll *a* than the green lettuce; moreover, the addition of Si fertilizer increased the concentration of chlorophyll *a* in the lettuce leaves by 37.5% (Table 2).

It should be stressed that the green lettuce accumulated more chlorophyll *a* when using Si fertilizer (71% more with sodium lighting), and the highest concentration of chlorophyll *a* was observed with LED lighting using 0.15% Si fertilizer (95% more than control under sodium lamps without the use of Si fertilizer).

For the red lettuce under sodium lighting, the addition of Si fertilizer to the solution caused an increase in the concentration of chlorophyll *a* (the best variant of the experiment), while under LED lighting the fertilizer contributed to a decrease in the concentration of the pigment. The data analysis showed that the specificity of the influence of factors on the concentration of chlorophyll *b* and carotenoids is similar to that of chlorophyll *a*, LSD = 0.2 (Table S2).

### 3.2. Effect of Fertilizer and Lighting on the Productivity of Lettuces after 30 Days of the Emergence of Mass Shoots

A significant positive effect of Si fertilizer was found on the 30th day of cultivation: the increase in fresh weight was 13.8%, LSD = 9.28 (Table S3), which indicates a delayed stress-protective effect of Si fertilizer even under light stress (Table 3).

Under the sodium lamps, the Si fertilizer caused a decrease in the fresh weight of lettuce of both varieties by 25.6%. The factors of lighting and nutrition did not have a significant effect on the dry mass, as well as on the leaf surface area, which means there is no loss in product quality. After light stress in chamber No. 1, by the 30th day, the plants had already adapted and significantly increased in terms of fresh and dry weight in a relatively short period of time. The same tendency was observed in the number of leaves in lettuce of both varieties (Table 3). The data show that the red lettuce is characterized by more intensive (14.2%) leaf formation than the green lettuce, which is a drumhead kind of lettuce, LSD = 1.51 (Table S3).

The best values of the sugar content for green lettuce were obtained under the sodium light with the addition of 0.15% of the Si fertilizer (12.1% higher sugar); however, for the red lettuce, the maximum sugar content was obtained under LED lighting also with the addition of Si fertilizer (by 8.2% compared to the control). In general, it appeared that the green lettuce accumulates more sugars by 9.9% (Table 4). The minimum concentration of nitrates for both varieties was observed under sodium lamps with Si fertilizer (14.8% lower); the concentrations in all other cases were within the allowed limits for this type of chemical compound (up to 4500 mg/kg). The use of LED and sodium lamps, as well as Si fertilizer, did not have a significant effect on the content of vitamin C and the basic pigments (Table S4).

### 3.3. Effect of Fertilizer and Lighting on the Productivity of Lettuces after 45 Days of the Emergence of Mass Shoots

**Figure 2:**
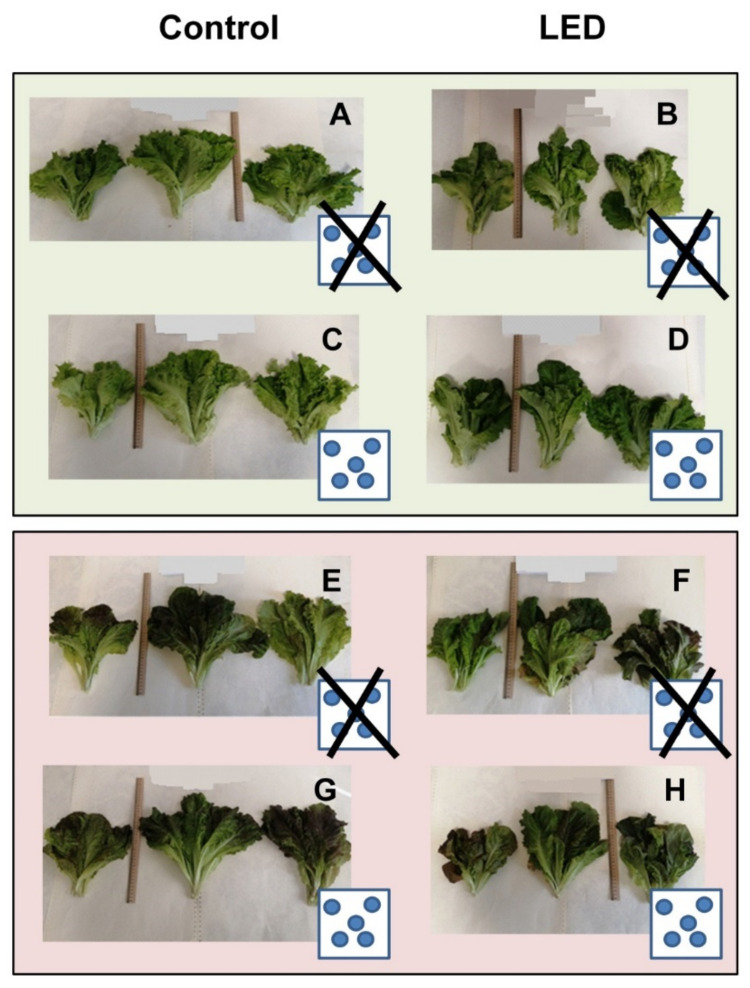
Comparison of the appearance of green (**A**–**D**) and red (**E**–**H**) lettuce on the 45th day of cultivation. Plants after sodium lamp illumination are on the left, after LED illumination on the right. (**C**,**D**,**G**,**H**) are the samples grown with Si fertilizer. (**A**,**B**,**E**,**F**) were grown without Si fertilizer.

The last sampling of the lettuce was done on the 45th day of cultivation when the plants correspond to marketable products (Figure 2). Considering the type of lighting and the variety of the lettuce as factors of statistical analysis, it was found that these factors had a significant effect on the fresh weight of the plants (Table 5 and Table 6). The distribution of the lettuce fresh weight group average of both varieties shows the advantage (about 25%) of sodium lighting over LEDs, LSD = 8.64 (Table S5). The green lettuce accumulates fresh mass more intensively than the red lettuce (by 22.3%).

The same advantage of sodium over LED lighting, about 12.2%, was revealed comparing the number of leaves of both varieties, LSD = 1.26 (Table S5). The red lettuce showed a tendency for more intensive leaf formation (19.4%).

Silicon fertilizer has had a significant effect on the content of chlorophyll *a*. The distribution of group averages of chlorophyll *a* concentration shows that the use of Si fertilizer increases the content of this pigment by 8.3%. It turned out that all three factors (type of lettuce, lighting, and Si fertilizer) have a significant effect on the content of chlorophyll *b*. The green lettuce accumulates 14% more chlorophyll *b* than that of the red lettuce. The largest accumulation of chlorophyll *b* in the green lettuce was observed under the LED illumination (21.9%), and for the red lettuce it was observed under sodium lamps (+18.5%). Addition of Si fertilizer increased the chlorophyll *b* concentration by 26.7% under the sodium lamp lighting, while under the LED lighting it was decreased by 15.5%. Finally, the use of Si fertilizer contributed to the accumulation of total chlorophyll in both varieties grown under sodium lamps by 26% (green) and 14.5% (red).

The range of the chlorophyll *a* to chlorophyll *b* ratio (which is about 2–3) for different combinations of the studied factors is due to the averaged samples taken from various tiers of plants (for heliophilous crops, this ratio is about three).

All three factors also significantly affect the concentration of carotenoids: the green lettuce accumulates 13% more of this pigment than the red lettuce (Table 6). Under the LED illumination, the carotenoids were accumulated by 13%, but the ratio of total chlorophyll to carotenoids is within the physiological norm (from three to eight), which means the plants adapted to the light conditions and do not have stress. The use of Si fertilizer caused an increase in the concentration of carotenoids for the green lettuce and a decrease for the red lettuce. In general, the concentration of nitrates in lettuces was affected by all factors, LSD = 0.95 (Table S6). The accumulation of nitrates was more than 5.5% under the LEDs. The addition of Si fertilizer reduced the concentration of nitrates by 9.3% under the sodium lamp lighting, but it was increased by 8.2% under the LEDs. For all cases, the total nitrates concentration was hundreds of times less than the threshold limit value on this product.

## 4. Discussion

Analysis of the results of this three-factor study allowed us to assess the influence of the biologically active Si fertilizer as well as the effect of different spectral compositions of LEDs in comparison with sodium lamps by using two climatic chambers (No. 1 and No. 2) on the biomass of freshly harvested lettuce of different varieties, red and green. Comparison of the biometric and biochemical data made it possible to draw certain conclusions on plant cultivation and development.

**Figure 3:**
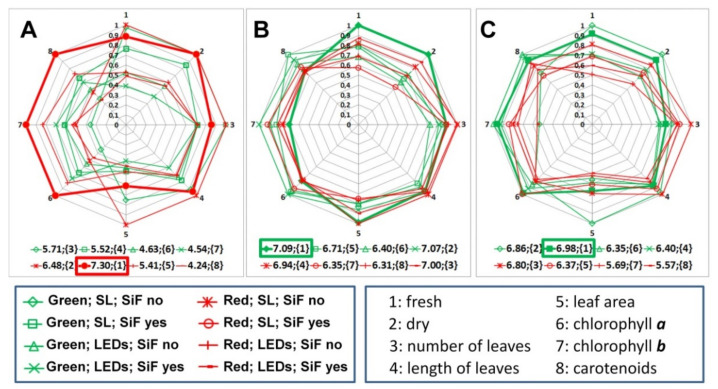
Comparison of the results of biometric and biochemical analysis for red and green lettuce after the 15th (**A**), 30th (**B**), and 45th (**C**) days of cultivation. The diagrams display the normalized parameters presented in Table 1, Table 2, Table 3, Table 4, Table 5 and Table 6 (eight parameters shown in the right panel at the bottom of the diagrams). The sets of parameters corresponding to a combination of three factors—type of lettuce, type of lighting, and the use of Si fertilizer—are connected by red or green lines with markers (the left panel at the bottom of the diagrams). The rank of each set of measured parameters is shown in curly brackets. Rectangular frames indicate the best combination of parameters obtained in the experiments.

Some earlier works claimed [45] that application of Si-containing fertilizer did not increase the lettuce yield. However, in our current and several recent studies [1,46], increased yield was observed as the positive effect of Si fertilizer, used as a microadditive to the nutrient solution, on the freshly harvested biomass of the green and red lettuces, grown under LED illumination on the 30th day of vegetation was established (Figure 3B). Table 7 contains the ranks for each combination of the three factors. One can see that for both types of lettuce and LED illumination on the 30th day, the second and the third ranks have samples grown with Si fertilizer. The increase in productivity of red lettuce for freshly harvested biomass was 26.6%, and for green lettuce 6.3%, while no loss of dry matter was observed.

It is interesting to note that when using hydroponic nutrient solution with Si [47], the lettuce plants showed a lower level of shoot dry matter, whereas the reverse effect [48] has been demonstrated too: the application of Si fertilizer via fertigation favored an increase in dry matter of lettuce. However, in our study a negative effect of Si fertilizer on the productivity of lettuce plants of both varieties on the 30th day of cultivation in chamber No. 2, under sodium lamp illumination, was noted (the 5th and the 7th ranks after the 30th day in Table 7). The productivity of green lettuce by estimating freshly harvested biomass decreased by 22.6%, while the red one decreased by 30.3% (without loss in dry matter percentage). Moreover, we observed an increase in the dry weight of lettuce (4.4%) when using the Si fertilizer in chamber No. 1 with LEDs’ irradiators on the 45 day.

We found that Si fertilizer helps to increase the leaf surface area and improve the marketable appearance of plants of both varieties (Figure 2). In addition, it increased the content of total chlorophyll, the maximum amount of which was observed when it was illuminated under sodium light.

There is a study [49] in which Si fertilizer was used in pots where lettuce was grown. In this case it did not cause significant differences in the leaf contents of nitrogen but increasing doses of silicate caused nitrogen deficiency. Our data and analysis revealed that the addition of Si fertilizer under sodium illumination reduced the concentration of nitrates by 9.3%; under the LEDs it was increased by 8.2%. Being grown under LED illumination, a more intense accumulation of nitrates was observed (about 5.5% more), which indicates that the synthesis of nitrogen-containing substances was intensified.

The effect of using Si fertilizer dramatically changed during the growing time: up to the 15th day of cultivation (Figure 3A) there was a sharp inhibition of the growth of lettuce plants of both varieties (the 7th and 8th ranks after the 15th day in Table 7); then, by the 30th day under LED lighting, the addition of Si fertilizer showed a stress-protective effect and had a positive influence in general. By the time of ripeness, the effect of using Si fertilizer was no longer observed. Therefore, the use of such a type of supplementary nutrition is advisable only when using LED illumination during the period of active plant growth (from the 10th day after germination).

It was recently reported [50] that the LED spectrum provided by the combination of far-red, deep-red, and blue LEDs is more favorable than sodium lamps for promoting the growth and nutrient uptake of plants. However, we found that the total productivity (3271.7 g) under the entire set of lettuce growth conditions (regardless of the lettuce variety) did not depend on the composition of the nutrient solution, but growth in chamber No. 2 (when illuminated with sodium lamps) was 35.5% higher than the plant productivity (2414.71 g) of the same lettuce varieties grown in chamber No. 1 (under the LED lighting).

## 5. Conclusions

Thus, considering the biometric and biochemical analysis, we can conclude that it makes sense to add Si fertilizer when growing red lettuce under LED lighting, while for the green lettuce the addition of such fertilizer is not useful. In the case of sodium lamp illumination, the best option for growing both varieties is without Si fertilizer.

## Figures and Tables

**Table 1:** Averaged biometric parameters of the aerial part of green and red lettuce (*Lactuca sativa* L.) under conditions of a regulated agroecosystem on the 15th day of cultivation. Values represent mean ± SE (*n* = 12). The different letters indicate significant differences among treatments mean non-significant differences among treatments according to Duncan’s test (*p* ≤ 0.05).

Lettuce	Lighting	Si	Plant Mass, g		Number of Leaves	Length of a Leaf, cm	Moisture, %	Leaf area, cm^2^
			Fresh	Dry				
Green	Sodium lamps	No	3.4 ± 0.8a	0.20 ± 0.02a	6.2 ± 0.4b	11.7 ± 0.9b	94.2 ± 0.2a	122.5 ± 8.6b
		Yes	2.7 ± 0.7c	0.17 ± 0.03	5.5 ± 0.4e	10.0 ± 1.2b	93.7 ± 0.4b	74.8 ± 4.4c
	LEDs	No	1.9 ± 0.4d	0.11 ± 0.01d	5.0 ± 0.4d	9.5 ± 1.1c	94.3 ± 0.5a	69.6 ± 7.2c
		Yes	1.4 ± 0.4e	0.08 ± 0.01e	4.7 ± 0.5	7.8 ± 0.8	94.1 ± 0.1a	58.6 ± 8.0c
Red	Sodium lamps	No	3.5 ± 0.9a	0.20 ± 0.03a	6.6 ± 0.5a	12.8 ± 1.1a	94.3 ± 0.2a	162.6 ± 10.9a
		Yes	3.1 ± 0.6b	0.20 ± 0.02b	6.2 ± 0.6b	12.1 ± 1.5a	93.8 ± 0.3b	99.6 ± 5.9bc
	LEDs	No	1.9 ± 0.4d	0.12 ± 0.01d	5.3 ± 0.7c	9.2 ± 1.1c	93.7 ± 0.5b	76.1 ± 3.0c
		Yes	1.7 ± 0.6d	0.11 ± 0.01d	5.2 ± 0.4c	9.1 ± 1.6c	93.6 ± 0.1b	67.2 ± 6.9c

**Table 2:** Average concentrations of photosynthetic pigments in green and red lettuce (*Lactuca sativa* L.) under conditions of a regulated agroecosystem on the 15th day of cultivation. Values represent mean ± SE (*n* = 12). The different letters indicate significant differences among treatments means non-significant differences among treatments according to Duncan’s test (*p* ≤ 0.05).

Lettuce	Lighting	Si	Chlorophyll *a*, mg/g	Chlorophyll *b*, mg/g	Carotenoids, mg/g
Green	Sodium lamps	No	1.04 ± 0.21e	0.34 ± 0.04e	0.38 ± 0.07f
		Yes	2.00 ± 0.04cd	0.59 ± 0.04d	0.69 ± 0.03b
	LEDs	No	1.65 ± 0.01d	0.59 ± 0.02d	0.52 ± 0.01cd
		Yes	2.27 ± 0.11c	0.67 ± 0.04c	0.63 ± 0.02c
Red	Sodium lamps	No	1.53 ± 0.01d	0.48 ± 0.03de	0.48 ± 0.02d
		Yes	2.98 ± 0.02a	0.96 ± 0.08a	1.04 ± 0.02a
	LEDs	No	2.45 ± 0.05b	0.80 ± 0.03b	0.75 ± 0.02b
		Yes	1.39 ± 0.34de	0.52 ± 0.09de	0.37 ± 0.15f

**Table 3:** Averaged biometric parameters of the aerial part of green and red lettuce (*Lactuca sativa* L.) under the conditions of a regulated agroecosystem on the 30th day of cultivation. Values represent mean ± SE (*n* = 12). The different letters indicate significant differences among treatments and ns means non-significant differences among treatments according to Duncan’s test (*p* ≤ 0.05).

Lettuce	Lighting	Si	Plant Mass, g		Number of Leaves	Length of a Leaf, cm	Moisture, %	Leaf Area, cm^2^	Photo Productivity, g/m^2^ Per Day
			Fresh	Dry					
Green	Sodium lamps	No	33.8 ± 15.0a	9.9 ± 0.9a	9.9 ± 2.1ab	22.6 ± 4.5ns	90.2 ± 0.1ns	1847.7 ± 7.6ns	5.5 ± 0.9a
		Yes	26.5 ± 11.8a	6.5 ± 0.5a	9.5 ± 1.4ab	20.0 ± 2.2ns	91.9 ± 1.0ns	1492.8 ± 8.1ns	6.3 ± 0.7a
	LEDs	No	25.6 ± 13.9a	6.0 ± 0.4a	8.1 ± 1.2b	21.1 ± 4.3ns	91.4 ± 0.9ns	1500.1 ± 5.6ns	6.2 ± 0.3a
		Yes	27.2 ± 8.8a	7.1 ± 0.5a	9.1 ± 1.8ab	22.8 ± 4.9ns	91.3 ± 1.2ns	1614.0 ± 9.1ns	6.1 ± 0.7a
Red	Sodium lamps	No	27.8 ± 11.9a	8.1 ± 0.3a	11.2 ± 2.6a	23.8 ± 5.1ns	90.3 ± 0.4ns	1871.2 ± 9.6ns	4.4 ± 0.2b
		Yes	19.3 ± 8.2b	5.3 ± 0.8b	9.9 ± 1.7ab	22.8 ± 2.3ns	90.9 ± 1.1ns	1402.3 ± 7.4ns	5.5 ± 0.8a
	LEDs	No	23.3 ± 13.4b	6.9 ± 0.7b	10.2 ± 2.3a	21.7 ± 3.3ns	90.1 ± 1.2ns	1428.4 ± 6.9ns	4.4 ± 0.9b
		Yes	29.4 ± 9.2a	8.8 ± 0.7a	11.1 ± 1.8a	23.2 ± 2.4ns	90.0 ± 0.9ns	1675.6 ± 8.4ns	6.1 ± 0.3a

**Table 4:** Averaged concentrations of sucrose, vitamin C, and nitrates in green and red lettuce (*Lactuca sativa* L.) under the conditions of a regulated agroecosystem on the 30th day of cultivation. Values represent mean ± SE (*n* = 12). The different letters indicate significant differences among treatments and ns means non-significant differences among treatments according to Duncan’s test (*p* ≤ 0.05).

Lettuce	Lighting	Si	Vitamin C, mg/100 g	Nitrate, mg/kg	Chlorophyll *a*, mg/g	Chlorophyll *b*, mg/g	Carotenoids, mg/g
Green	Sodium lamps	No	15.1 ± 0.1ns	5.1 ± 0.1ab	2.2 ± 0.5ns	0.6 ± 0.1ns	0.7 ± 0.2ns
		Yes	14.9 ± 0.2ns	5.0 ± 0.4ab	2.6 ± 0.8ns	0.8 ± 0.2ns	0.9 ± 0.3ns
	LEDs	No	15.0 ± 0.1ns	5.3 ± 0.1a	2.5 ± 0.6ns	0.8 ± 0.2ns	0.8 ± 0.2ns
		Yes	14.9 ± 0.1ns	5.3 ± 0.1a	2.7 ± 0.5ns	0.9 ± 0.2ns	0.8 ± 0.2ns
Red	Sodium lamps	No	14.9 ± 0.03ns	5.7 ± 0.4a	2.1 ± 0.9ns	0.7 ± 0.3ns	0.7 ± 0.3ns
		Yes	15.0 ± 0.03ns	4.3 ± 0.1b	2.5 ± 0.4ns	0.8 ± 0.1ns	0.7 ± 0.1ns
	LEDs	No	14.9 ± 0.1ns	4.9 ± 0.2b	2.2 ± 0.6ns	0.7 ± 0.2ns	0.7 ± 0.2ns
		Yes	14.9 ± 0.1ns	5.4 ± 0.1a	2.1 ± 0.1ns	0.6 ± 0.0ns	0.7 ± 0.04ns

**Table 5:** Averaged biometric parameters of the aerial part of green and red lettuce (*Lactuca sativa* L.) under conditions of a regulated agroecosystem on the 45th day of cultivation. Values represent mean ± SE (*n* = 12). The different letters indicate significant differences among treatments and ns means non-significant differences among treatments according to Duncan’s test (*p* ≤ 0.05).

Lettuce	Lighting	Si	Plant Mass, g		Number of Leaves	Length of a leaf, cm	Moisture, %	Leaf Area, cm^2^	Photo Productivity, g/m^2^ per day
			Fresh	Dry					
Green	Sodium lamps	No	81.1 ± 21.7a	3.3 ± 0.1a	13.8 ± 1.5ab	30.5 ± 3.5a	95.9 ± 0.1a	4661.2 ± 6.5a	1.0 ± 0.7ns
		Yes	74.3 ± 19.3a	3.0 ± 0.1a	12.8 ± 1.4ab	27.7 ± 2.9b	95.9 ± 0.1a	3135.6 ± 5.2a	0.4 ± 0.2ns
	LEDs	No	57.6 ± 21.2b	2.3 ± 0.1b	11.9 ± 2.3b	27.3 ± 3.7b	96.0 ± 0.1a	2525.5 ± 5,7b	1.1 ± 0.4ns
		Yes	57.6 ± 18.8b	2.6 ± 0.1b	11.6 ± 1.1b	26.8 ± 3.0b	95.5 ± 0.2b	2748.4 ± 4.5b	1.2 ± 0.2ns
Red	Sodium lamps	No	65.5 ± 25.8b	2.8 ± 0.1b	17.2 ± 5.1a	31.4 ± 3.7a	95.7 ± 0.1b	3253.4 ± 5.7a	0.8 ± 0.2ns
		Yes	55.7 ± 14.9b	2.4 ± 0.1b	15.2 ± 2.7a	29.0 ± 2.8a	95.6 ± 0.2b	2825.9 ± 8.1a	0.9 ± 0.2ns
	LEDs	No	41.1 ± 19.8c	1.9 ± 0.04c	14.7 ± 3.7ab	25.9 ± 4.1c	95.3 ± 0.1c	2413.3 ± 6.6b	1.5 ± 0.2ns
		Yes	48.2 ± 20.7b	2.3 ± 0.1b	14.8 ± 3.6ab	24.3 ± 3.0c	95.2 ± 0.1c	2299.1 ± 7.0b	1.3 ± 0.3ns

**Table 6:** Average concentrations of chlorophyll *a* and *b*, carotenoids, and nitrates in green and red lettuce (*Lactuca sativa* L.) under conditions of a regulated agroecosystem on the 45th day of cultivation. Values represent mean ± SE (*n* = 12). The different letters indicate significant differences among treatments means non-significant differences among treatments according to Duncan’s test (*p* ≤ 0.05).

Lettuce	Lighting	Si	Nitrate, mg/kg	Chlorophyll *a*, mg/g	Chlorophyll *b*, mg/g	Carotenoids, mg/g
Green	Sodium lamps	No	5.13 ± 0.08b	2.09 ± 0.07b	0.71 ± 0.05c	0.74 ± 0.12b
		Yes	5.16 ± 0.43b	2.50 ± 0.21a	1.27 ± 0.15a	0.92 ± 0.01ab
	LEDs	No	5.25 ± 0.02b	2.18 ± 0.41b	1.33 ± 0.22a	1.01 ± 0.04a
		Yes	5.5 ± 0.12ab	2.33 ± 0.20a	1.23 ± 0.05a	0.97 ± 0.07a
Red	Sodium lamps	No	5.58 ± 0.16a	2.07 ±0.20b	1.06 ± 0.11b	0.84 ± 0.02ab
		Yes	4.55 ± 0.02c	2.54 ± 0.09a	1.12 ± 0.04b	0.70 ± 0.06b
	LEDs	No	5.11 ± 0.07b	1.99 ± 0.11b	1.00 ± 0.02b	0.88 ± 0.01ab
		Yes	5.78 ± 0.06a	2.08 ± 0.05b	0.74 ± 0.03c	0.79 ± 0.02b

**Table 7:** Rank of each combination of factors (plants, lighting, Si fertilizer (SiF)) after the 15th, 30th, and 45th days of cultivation and the total ranks (*Σ*).

Factors	Rank after			*Σ*
	the 15th day	the 30th day	the 45th day	
Green; SL; SiF no	3	1	2	6
Green; SL; SiF yes	4	5	1	10
Green; LEDs; SiF no	6	6	6	18
Green; LEDs; SiF yes	7	2	4	13
Red; SL; SiF no	2	4	3	9
Red; SL; SiF yes	1	7	5	13
Red; LEDs; SiF no	5	8	7	20
Red; LEDs; SiF yes	8	3	8	19

## Data Availability

No additional data available.

## Supplementary Materials

The following are available online at https://www.mdpi.com/article/10.3390/plants10071287/s1, Table S1. LCD values for biometric parameters of the aerial part of green and red lettuce (*Lactuca sativa* L.) under conditions of a regulated agroecosystem on the 15th day of cultivation; Table S2. LCD values for concentrations of photosynthetic pigments in green and red lettuce (*Lactuca sativa* L.) under conditions of a regulated agroecosystem on the 15th day of cultivation; Table S3. LCD values for biometric parameters of the aerial part of green and red lettuce (*Lactuca sativa* L.) under the conditions of a regulated agroecosystem on the 30th day of cultivation; Table S4. LCD values for concentrations of sucrose, vitamin C, and nitrates in green and red lettuce (*Lactuca sativa* L.) under the conditions of a regulated agroecosystem on the 30th day of cultivation; Table S5. LCD values for biometric parameters of the aerial part of green and red lettuce (*Lactuca sativa* L.) under the conditions of a regulated agroecosystem on the 45th day of cultivation; Table S6. LCD values for concentrations of chlorophyll a and b, carotenoids, and nitrates in green and red lettuce (*Lactuca sativa* L.) under the conditions of a regulated agroecosystem on the 45th day of cultivation.

## References

[1] Virsile A., Brazaityte A., Vastakaite-Kairiene V., Miliauskiene J., Jankauskiene J., Novickovas A., Lauzike K., Samuoliene G. (2020). The distinct impact of multi-color LED light on nitrate, amino acid, soluble sugar and organic acid contents in red and green leaf lettuce cultivated in controlled environment. Food Chem..

[2] Camejo D., Frutos A., Mestre T.C., Piñero M.D., Rivero R.M., Martínez V. (2020). Artificial light impacts the physical and nutritional quality of lettuce plants. Hortic. Environ. Biotechnol..

[3] Davis P.A., Burns C. (2016). Photobiology in protected horticulture. Food Energy Secur..

[4] Najera C., Urrestarazu M. (2019). Effect of the Intensity and Spectral Quality of LED Light on Yield and Nitrate Accumulation in Vegetables. Hortscience.

[5] Shevela D., Pishchainikov R.Y., Eichacker L.A., Govindjee (2013). Stress Biology of Cyanobacteria: Molecular Mechanism to Cellular Responses. Oxygenic Photosynthesis in Cyanobacteria.

[6] Pishchalnikov R.Y., Razjivin A.P. (2014). From localized excited states to excitons: Changing of conceptions of primary photosynthetic processes in the twentieth century. Biochem. Mosc..

[7] Gudkov S.V., Andreev S.N., Barmina E.V., Bunkin N.F., Kartabaeva B.B., Nesvat A.P., Stepanov E.V., Taranda N.I., Khramov R.N., Glinushkin A.P. (2017). Effect of visible light on biological objects: Physiological and pathophysiological aspects. Phys. Wave Phenom..

[8] Kim H.H., Goins G.D., Wheeler R.M., Sager J.C. (2004). Green-light supplementation for enhanced lettuce growth under red- and blue-light-emitting diodes. Hortscience.

[9] Hernandez R., Kubota C. (2016). Physiological responses of cucumber seedlings under different blue and red photon flux ratios using LEDs. Environ. Exp. Bot..

[10] Johkan M., Shoji K., Goto F., Hashida S., Yoshihara T. (2010). Blue Light-emitting Diode Light Irradiation of Seedlings Improves Seedling Quality and Growth after Transplanting in Red Leaf Lettuce. Hortscience.

[11] Huche-Thelier L., Crespel L., Le Gourrierec J., Morel P., Sakr S., Leduc N. (2016). Light signaling and plant responses to blue and UV radiations-Perspectives for applications in horticulture. Environ. Exp. Bot..

[12] Bhuiyan R., van Iersel M.W. (2021). Only Extreme Fluctuations in Light Levels Reduce Lettuce Growth Under Sole Source Lighting. Front. Plant Sci..

[13] Gherghina E., Luta G., Dobrin E., Draghici E.M., Balan D., Sanmartin A.M. (2020). Biochemical changes under artificial led lighting in some *Lactuca sativa* L. varieties. Agrolife Sci. J..

[14] Rizwan M., Rehman M.Z.U., Ali S., Abbas T., Maqbool A., Bashir A. (2019). Biochar Is a Potential Source of Silicon Fertilizer: An Overview.

[15] Tripathi P., Na C.I., Kim Y. (2021). Effect of silicon fertilizer treatment on nodule formation and yield in soybean (*Glycine max* L.). Eur. J. Agron..

[16] Stephano M.F., Geng Y.H., Cao G.J., Wang L.C., Meng W., Zhang M.L. (2021). Effect of Silicon Fertilizer and Straw Return on the Maize Yield and Phosphorus Efficiency in Northeast China. Commun. Soil Sci. Plant Anal..

[17] Huang H.L., Rizwan M., Li M., Song F.R., Zhou S.J., He X., Ding R., Dai Z.H., Yuan Y., Cao M.H. (2019). Comparative efficacy of organic and inorganic silicon fertilizers on antioxidant response, Cd/Pb accumulation and health risk assessment in wheat (*Triticum aestivum* L.). Environ. Pollut..

[18] Fan Y., Shen W.Y., Cheng F.Q. (2018). Reclamation of two saline-sodic soils by the combined use of vinegar residue and silicon-potash fertiliser. Soil Res..

[19] Franca A.A., Schultz J., Borges R., Wypych F., Mangrich A.S. (2017). Rice Husk Ash as Raw Material for the Synthesis of Silicon and Potassium Slow-Release Fertilizer. J. Braz. Chem. Soc..

[20] Eneji A.E., Inanaga S., Muranaka S., Li J., Hattori T., An P., Tsuji W. (2008). Growth and nutrient use in four grasses under drought stress as mediated by silicon fertilizers. J. Plant Nutr..

[21] Debona D., Rodrigues F.A., Datnoff L.E., Leach J.E., Lindow S.E. (2017). Silicon’s Role in Abiotic and Biotic Plant Stresses. Annual Review of Phytopathology.

[22] Houben D., Sonnet P., Cornelis J.T. (2014). Biochar from Miscanthus: A potential silicon fertilizer. Plant Soil.

[23] Ma J.F., Yamaji N., Mitani-Ueno N. (2011). Transport of silicon from roots to panicles in plants. Proc. Jpn. Acad. Ser. B-Phys. Biol. Sci..

[24] Huang C.P., Wang L., Gong X.Q., Huang Z.T., Zhou M.R., Li J., Wu J.S., Chang S.X., Jiang P.K. (2020). Silicon fertilizer and biochar effects on plant and soil PhytOC concentration and soil PhytOC stability and fractionation in subtropical bamboo plantations. Sci. Total. Environ..

[25] Currie H.A., Perry C.C. (2007). Silica in plants: Biological, biochemical and chemical studies. Ann. Bot..

[26] Cuong T.X., Ullah H., Datta A., Hanh T.C. (2017). Effects of Silicon-Based Fertilizer on Growth, Yield and Nutrient Uptake of Rice in Tropical Zone of Vietnam. Rice Sci..

[27] Miyake Y., Takahashi E. (1983). Effect of silicon on the growth of solution-cultured cucumber plant. Soil Sci. Plant Nutr..

[28] Seebold K.W., Kucharek T.A., Datnoff L.E., Correa-Victoria F.J., Marchetti M.A. (2001). The influence of silicon on components of resistance to blast in susceptible, partially resistant, and resistant cultivars of rice. Phytopathology.

[29] Stevens W., Rhine M., Vories E. (2017). Effect of Irrigation and Silicon Fertilizer on Total Rice Grain Arsenic Content and Yield. Crop. Forage Turfgrass Manag..

[30] Yu T.H., Peng Y.Y., Lin C.X., Qin J.H., Li H.S. (2016). Application of iron and silicon fertilizers reduces arsenic accumulation by two Ipomoea aquatica varities. J. Integr. Agric..

[31] Bian Z.H., Lei B., Cheng R.F., Wang Y., Li T., Yang Q.C. (2020). Selenium distribution and nitrate metabolism in hydroponic lettuce (*Lactuca sativa* L.): Effects of selenium forms and light spectra. J. Integr. Agric..

[32] Yan Z.N., He D.X., Niu G.H., Zhou Q., Qu Y.H. (2019). Growth, Nutritional Quality, and Energy Use Efficiency of Hydroponic Lettuce as Influenced by Daily Light Integrals Exposed to White versus White Plus Red Light-emitting Diodes. Hortscience.

[33] Yan Z.N., He D.X., Niu G.H., Zhai H. (2019). Evaluation of growth and quality of hydroponic lettuce at harvest as affected by the light intensity, photoperiod and light quality at seedling stage. Sci. Hortic..

[34] Virsile A., Brazaityte A., Vastakaite-Kairiene V., Miliauskiene J., Jankauskiene J., Novickovas A., Samuoliene G. (2019). Lighting intensity and photoperiod serves tailoring nitrate assimilation indices in red and green baby leaf lettuce. J. Sci. Food Agric..

[35] Naznin M.T., Lefsrud M., Gravel V., Azad M.O.K. (2019). Blue Light added with Red LEDs Enhance Growth Characteristics, Pigments Content, and Antioxidant Capacity in Lettuce, Spinach, Kale, Basil, and Sweet Pepper in a Controlled Environment. Plants.

[36] Meng Q.W., Kelly N., Runkle E.S. (2019). Substituting green or far-red radiation for blue radiation induces shade avoidance and promotes growth in lettuce and kale. Environ. Exp. Bot..

[37] Shevtsova L.P., Shyurova N.A., Bashinskaya O.S., Toigildin A.L., Toigildina I.A. (2016). Practices of Raising the Cropping Power of Green Large Seed Lentil in the Volga Region Steppe. Res. J. Pharm. Biol. Chem. Sci..

[38] Chung H.Y., Chang M.Y., Wu C.C., Fang W. (2018). Quantitative Evaluation of Electric Light Recipes for Red Leaf Lettuce Cultivation in Plant Factories. Horttechnology.

[39] Spadafora N.D., Cocetta G., Ferrante A., Herbert R.J., Dimitrova S., Davoli D., Fernandez M., Patterson V., Vozel T., Amarysti C. (2020). Short-Term Post-Harvest Stress that Affects Profiles of Volatile Organic Compounds and Gene Expression in Rocket Salad during Early Post-Harvest Senescence. Plants.

[40] Lee H.J., Chun J.H., Kim S.J. (2017). Effects of Pre Harvest Light Treatments (LEDs, Fluorescent Lamp, UV-C) on Glucosinolate Contents in Rocket Salad (*Eruca sativa*). Hortic. Sci. Technol..

[41] Jin J., Koroleva O.A., Gibson T., Swanston J., Magan J., Zhang Y., Rowland I.R., Wagstaff C. (2009). Analysis of Phytochemical Composition and Chemoprotective Capacity of Rocket (Eruca sativa and Diplotaxis tenuifolia) Leafy Salad Following Cultivation in Different Environments. J. Agric. Food Chem..

[42] Santamaria P., Gonnella M., Elia A., Parente A., Serio F., Maloupa E., Gerasopoulos D. (2001). Ways of reducing rocket salad nitrate content. Proceedings of the International Symposium on Growing Media and Hydroponics.

[43] Karayannis M.I. (1975). Kinetic determination of ascorbic acid by the 2,6-dichlorophenolindophenol reaction with a stopped-flow technique. Anal. Chim. Acta.

[44] Turhan A., Kuscu H., Ozmen N., Serbeci M.S., Demir A.O. (2014). Effect of different concentrations of diluted seawater on yield and quality of lettuce. Chil. J. Agric. Res..

[45] Ferreira R.L.F., Souza R.J., de Carvalho J.G., Neto S.E.D., Yuri J.E. (2009). Evaluation lettuce cultivars fertilizer with silifertil (R). Rev. Caatinga.

[46] Esmaili M., Mashal M., Aliniaeifard S., Urrestarazu M., Carrillo F.F. (2021). Impact of Silicon on Chemical Properties of Drainage Water from Lettuce Following Determination of Proper Cultivar and Light Spectrum. Commun. Soil Sci. Plant Anal..

[47] Luz J., Guimaraes S.T.M.R., Korndörfer G.H. (2006). Produção hidropônica de alface em solução nutritiva com e sem silício. Hortic. Bras..

[48] De Souza R.S., Rezende R., de Freitas P.S.L., Goncalves A.C.A., Rezende G.S. (2015). Dry matter production and macronutrient leaf composition in lettuce under fertigation with nitrogen, potassium and silicon. Rev. Bras. Eng. Agric. Ambient..

[49] Ferreira R.L.F., de Souza R.J., de Carvalho J.G., de Araujo S.E.D., Mendonca V., Wadt P.G.S. (2010). Evaluation of lettuce cultivars fertilized with calcium silicate in greenhouse. Cienc. Agrotecnol..

[50] Pinho P., Jokinen K., Halonen L. (2017). The influence of the LED light spectrum on the growth and nutrient uptake of hydroponically grown lettuce. Lighting Res. Technol..

