# Supplementary material for: The Effect of Plant Growth Compensation by Adding Silicon-Containing Fertilizer under Light Stress Conditions
Source: Plants (Basel). 2021 Jun 24;10(7):1287. doi: 10.3390/plants10071287 (PMC8308918; doi:10.3390/plants10071287)
Supplement: Supplementary file 1 [file plants-10-01287-s001.zip › plants-1222018-supplementary.pdf]

# Supplementary Materials

**Table S1.** LCD values for biometric parameters of the aerial part of green and red lettuce (*Lactuca sativa L.*) under conditions of a regulated agroecosystem on the 15 th day of cultivation.

| Lighting (A) | Si (B) | Lettuce (C) | Plant mass, g | | Number of leaves | Length of a leaf, cm | Moisture, % | Leaf area, cm$^2$ |
|---|---|---|---|---|---|---|---|---|
| | | | Fresh | dry | | | | |
| **LSD ($p$ < 0.05) A** | | | **0.26** | **0.05** | **0.21** | **0.5** | **-** | 21.61 |
| LSD ($p$ < 0.05) B | | | 0.26 | 0.05 | 0.21 | 0.5 | - | 21.61 |
| LSD ($p$ < 0.05) C | | | - | - | 0.21 | 0.5 | - | - |
| LSD ($p$ < 0.05) AB | | | - | - | - | - | - | 40.75 |
| LSD ($p$ < 0.05) AC | | | - | - | - | - | 0.65 | - |
| LSD ($p$ < 0.05) BC | | | - | - | - | 0.93 | - | - |
| LSD ($p$ < 0.05) ABC | | | - | - | - | - | - | - |

**Table S2.** LCD values for concentrations of photosynthetic pigments in green and red lettuce (*Lactuca sativa L.*) under conditions of a regulated agroecosystem on the 15 th day of cultivation.

| Lighting (A) | Si (B) | Lettuce (C) | Chlorophyll *a*, mg/g | Chlorophyll *b*, mg/g | Carotenoids, mg/g |
|---|---|---|---|---|---|
| LSD ($p$ < 0.05) A | | | - | - | - |
| LSD ($p$ < 0.05) B | | | 0.15 | 0.06 | 0.06 |
| LSD ($p$ < 0.05) C | | | 0.15 | 0.06 | 0.06 |
| LSD ($p$ < 0.05) AB | | | 0.29 | 0.11 | 0.12 |
| LSD ($p$ < 0.05) AC | | | 0.29 | 0.11 | 0.12 |
| LSD ($p$ < 0.05) BC | | | 0.29 | - | - |
| LSD ($p$ < 0.05) ABC | | | 0.56 | 0.20 | 0.23 |

**Table S3.** LCD values for biometric parameters of the aerial part of green and red lettuce (*Lactuca sativa L.*) under the conditions of a regulated agroecosystem on the 30 th day of cultivation.

| Lighting (A) | Si (B) | Lettuce (C) | Plant mass, g | | Number of leaves | Length of a leaf, cm | Moisture, % | Leaf area, cm$^2$ | Photo productivity, g/m$^2$ per day |
|---|---|---|---|---|---|---|---|---|---|
| | | | Fresh | Dry | | | | | |
| **LSD ($p$ < 0.05) A** | | | **-** | **-** | **-** | **-** | **-** | **-** | |
| LSD ($p$ < 0.05) B | | | - | - | - | - | - | - | |
| LSD ($p$ < 0.05) C | | | - | - | 0.81 | - | - | - | |
| LSD ($p$ < 0.05) AB | | | 9.28 | - | 1.51 | - | - | - | 0.7 |
| LSD ($p$ < 0.05) AC | | | - | - | - | - | - | - | - |
| LSD ($p$ < 0.05) BC | | | - | - | - | - | - | - | - |
| LSD ($p$ < 0.05) ABC | | | - | - | - | - | - | - | - |

**Table S4.** LCD values for concentrations of sucrose, vitamin C, and nitrates in green and red lettuce (*Lactuca sativa L.*) under the conditions of a regulated agroecosystem on the 30 th day of cultivation.

| Lighting (A) | Si (B) | Lettuce (C) | Vitamin C, mg/100 g | Nitrate, mg/kg | Chlorophyll *a*, mg/g | Chlorophyll *b*, mg/g | Carotenoids, mg/g |
|---|---|---|---|---|---|---|---|
| LSD ($p$ < 0.05) A | | | - | - | - | - | - |
| LSD ($p$ < 0.05) B | | | - | - | - | - | - |
| LSD ($p$ < 0.05) C | | | - | - | - | - | - |
| LSD ($p$ < 0.05) AB | | | - | 0.67 | - | - | - |
| LSD ($p$ < 0.05) AC | | | - | - | - | - | - |
| LSD ($p$ < 0.05) BC | | | - | - | - | - | - |
| LSD ($p$ < 0.05) ABC | | | - | 1.28 | - | - | - |

**Table S5.** LCD values for biometric parameters of the aerial part of green and red lettuce (*Lactuca sativa L.*) under the conditions of a regulated agroecosystem on the 45 th day of cultivation.

| Lighting (A) | Si (B) | Lettuce (C) | Plant mass, g | | Number of leaves | Length of a leaf, cm | Moisture, % | Leaf area, $cm^2$ | Photo productivity, $g/m^2$ per day |
|---|---|---|---|---|---|---|---|---|---|
| | | | Fresh | Dry | | | | | |
| **LSD ($p < 0.05$) A** | | | **8.64** | **-** | **1.26** | **1.41** | **0.11** | **638.53** | - |
| LSD ($p < 0.05$) B | | | - | - | - | 1.41 | - | - | - |
| LSD ($p < 0.05$) C | | | 8.64 | - | 1.26 | - | 0.11 | - | - |
| LSD ($p < 0.05$) AB | | | - | - | - | - | 0.21 | - | - |
| LSD ($p < 0.05$) AC | | | - | - | - | 2.64 | 0.21 | - | - |
| LSD ($p < 0.05$) BC | | | - | - | - | - | - | - | - |
| LSD ($p < 0.05$) ABC | | | - | - | - | - | - | - | - |

**Table S6.** LCD values for concentrations of chlorophyll *a* and *b*, carotenoids, and nitrates in green and red lettuce (*Lactuca sativa L.*) under the conditions of a regulated agroecosystem on the 45 th day of cultivation.

| Lighting (A) | Si (B) | Lettuce (C) | Nitrate, mg/kg | Chlorophyll *a*, mg/g | Chlorophyll *b*, mg/g | Carotenoids, mg/g |
|---|---|---|---|---|---|---|
| LSD ($p < 0.05$) A | | | 0.26 | - | - | 0.06 |
| LSD ($p < 0.05$) B | | | - | 0.21 | - | - |
| LSD ($p < 0.05$) C | | | - | - | 0.11 | 0.06 |
| LSD ($p < 0.05$) AB | | | 0.50 | - | 0.21 | - |
| LSD ($p < 0.05$) AC | | | - | - | 0.21 | - |
| LSD ($p < 0.05$) BC | | | - | - | 0.21 | 0.11 |
| LSD ($p < 0.05$) ABC | | | 0.95 | - | - | 0.22 |
